# Pulse oximetry as a screening test for congenital heart disease in newborns

**DOI:** 10.34763/jmotherandchild.20222601.d-21-00033

**Published:** 2022-07-20

**Authors:** Dalwinder Janjua, Japna Singh, Amit Agrawal

**Affiliations:** Department of Neonatology, Al Jalila Children’s Hospital, Dubai, UAE; International Modern Hospital, Dubai, UAE; Department of Paediatrics, Gandhi Medical College, Bhopal, Madhya Pradesh, India

**Keywords:** congenital heart disease (CHD), pulse oximetry, physical examination, echocardiography

## Abstract

**Background:**

Congenital heart disease (CHD) can be fatal if not diagnosed at the early phases of life. Available diagnostic tools for screening critical CHD are mostly invasive and costly.

**Aim:**

The current study aimed to validate the use of pulse oximetry as a non-invasive and cost-effective tool to screen critical CHD.

**Material and methods:**

This observational study was conducted in a tertiary care teaching institute. A total of 1,082 asymptomatic term neonates (aged 2–24 h) were screened by pulse oximetry and clinical examination for the detection of critical CHD. Neonates with abnormal pulse oximetry and clinical examination findings were subjected to confirm the presence of CHD.

**Results:**

The incidence of critical CHD in asymptomatic newborns was found to be 0.5% (5/1000 live births). Echocardiography confirmed five cases of critical CHD. Pulse oximetry alone could detect 80%, and clinical examination alone could detect 60% of the CHD cases, while combining both methods gave 100% detection rate.

**Conclusion:**

Pulse oximetry is a simple, cost-effective, and reliable tool to diagnose critical CHD. In majority of the newborns who have not undergone fetal echocardiography, the underlying critical CHD can be missed, and in such cases, pulse oximetry screening offers an effective way to minimise the undiagnosed discharge risk.

## Introduction

Congenital heart disease (CHD) is defined as the structural, functional, or positional defect of the heart, present at birth but that may manifest itself at any time after birth or may not manifest at all [1]. The incidence of moderate to severe structural CHD in live-born infants is 6–8/1,000 live births in different areas round the world, and this has remained constant over the years [2, 3, 4]. The prevalence of critical CHD found in India shows a wide variation from 3.9–26.4/1,000 live births [5]. Critical CHD accounts for the highest mortality rate compared with any other congenital disorders [6, 7, 8]. In India, approximately 10% of present infant mortality accounted for the critical CHD alone [5]. Usually, most of the CHD can be treated successfully; however, exceptional clinical conditions may often result in severe disorders such as hypoplastic left heart syndrome (HLHS) [9,10] and transposition of the great arteries (TGA) [11]. Early diagnosis of CHD is important for to ensure its management and to avoid morbidity and mortality in future phases of life [7].

Among the customary practices of diagnostic tools, prenatal tools include ultrasonography and fetal echocardiography (Echo) [12] while postnatal screening relies on clinical examination findings such as murmurs, abnormal BP, abnormal electrocardiography (ECG), and abnormal X-rays. However, this may not help diagnose critical CHD on the first day of life [13]. The prevalence of murmurs in neonates varies from <1% to >50% [14]. Murmurs heard in newborns are often physiological or may be associated with structural heart disease [14]. On the other hand, serious heart defects may be present with little or no murmur [15]. Echo is important not only in the diagnostic evaluation of critical CHD but also in the overall assessment of the cardiovascular system in disorders unique to the newborn infant [16]. M-Mode, two-dimensional, and doppler echo are used to provide more meaningful information than the conventional ECG and X-ray. However, the high cost and limited availability, especially in resource-limited settings, are the major limitations of echo. Other drawbacks include inadequate visualisation of structures due to lung disease, air trapping, and inadequate doppler signal for the velocity measurement. Neonatologists are usually trained to detect Patent Ductus Arteriosus (PDA) but do not have the expertise to pick up other CHD by Echo [17, 18].

In the last decade, pulse oximetry (POx) has been suggested as a diagnostic tool to detect critical CHD, and it has been made compulsory in a few states in the United States [19, 20, 21]. A common feature of many forms of critical CHD is hypoxemia, which can be monitored using a pulse oximeter. Often mild cyanosis is not visible clinically; hence, the POx can be used as an easy, quick, non-invasive, and cost-effective tool to detect this cyanosis in suspected cases of CHD [19]. This offers an effective way to minimise the risk of discharge of undiagnosed cases [21]. Pulse oximeters are easy to use and can be used by the nursing staff also. The heart rate is also displayed, and the saturation of all four limbs can be determined [19, 20]. However, POx does not detect left-to-right shunt or other critical CHD not affecting oxygenation. Defects like coarctation of the aorta (COA) and total anomalous pulmonary venous return (TAPVR) are very likely to be missed by POx [22]. The current study aimed to assess the effectiveness and usefulness of POx for a timely diagnosis of critical CHD.

## Material and methods

This prospective observational study was undertaken over one year and one month in the postnatal ward of a tertiary care teaching institution. The study population comprised all "asymptomatic term babies" born in the maternity ward of our medical college. Babies born preterm (< 37 weeks) or those with birth asphyxia, sepsis, respiratory distress, convulsions, or major congenital abnormalities were not included in the study. Those fulfilling the following inclusion criteria were enrolled for the study: babies with weight equal to or greater than 2,500 grams, gestation equal to more than 37 weeks but less than 42 weeks, and age greater than two hours of life. A detailed history and clinical examination, specifically the antenatal history of drugs and family history of CHD were performed on all the neonates included. A cardiovascular examination was performed as well.

Prospective screening of post-ductal oxygen saturation (SpO_2_) was conducted on either foot using a pulse oximeter (L&T Multipara monitor Planet 40 Nellcor oximax SpO2 module with SpO_2_ dura sensor DYS 100A neonatal Y probe), and functional saturation of all newborns was recorded. The probe was fixed on either foot using Velcro straps and results were recorded in a quiet resting state, once a consistent pulse waveform was established. When the pulse oximeter showed a good pulse wave, the maximum value of SpO_2_ was recorded. The infant’s age at screening, sex, and delivery mode (Caesarean section or vaginal) were noted.

We used postductal SpO_2_ because measurements of SpO_2_ in the lower extremity is slightly lower than in the upper extremity in the newborn at 24 hours of life owing to shunting at the level of the *ductus arteriosus* [23]. In general, the mean difference between the SpO_2_ in the upper and lower extremities is < 1%; however, some newborns with critical CHD may have a more profound difference in SpO_2_ between the upper and lower body. For example, neonates with some forms of left obstructive heart lesions, such as critical COA, in which the *ductus arteriosus* supplies a portion of the systemic flow, may have lower Spo_2_ readings in the legs than in the arm [24].

When postductal SpO_2_ was less than 95%, the baby was provisionally considered to be screening positive. Babies with SpO_2_ less than 90% were considered abnormal and were subjected to echo. For those with SpO_2_ between 90%–94%, repeated measurements were performed after one hour, and babies with more than two repeated positive measurements (< 95%) were subjected to echo.

Detailed cardiovascular examination was performed on all the participants in 2–24 hours of life including four-limb blood pressure measurements, palpation of femoral pulses and other peripheral pulses, auscultation of heart sounds, murmurs or other abnormal heart sounds, hepatomegaly, and edema [25]. In addition, dysmorphism, colour, respiratory rate, and capillary refilling time were also assessed [26]. The intensity of the murmur was graded from grade 1 to grade 6. Newborns with innocent murmurs (short, systolic, not very loud, < grade 3) were considered normal [27]. In infants with COA, the femoral pulse may be normal in the first few days of life while the ductus is still open [28].

Chest radiography and echo were performed in all the neonates with positive POx screening (SpO_2_ < 95%) and those suspected to have CHD on clinical examination. Doppler echo was done with Biosound Esaote /P (Lab 5) with a paediatric probe 7.5-mHz transducer. Critical CHD was defined as any cardiac lesion from which infants die or require surgery or cardiac catheterisation within the first 28 days of life to prevent death or severe end-organ damage, according to the American Heart Association and American Academy of Pediatrics guidelines [19]. Both cyanotic and acyanotic CHDs were included in the present study.

Figure 1 shows the overall workflow of the study. The study was approved by the local Ethics Committee. Information regarding the study was presented to the parents, and informed consent was obtained.

**Statistical analysis:** The data were collected in a predesigned structured form and were compiled using Microsoft Excel. Categorial data were expressed as numbers and percentages whereas numerical data were expressed as mean and standard deviation. The analysis was carried out using SPSS Version 16 statistical software.

## Results

A total of 1,082 neonates were included in the study. No mother had a history of substance abuse, hereditary disorder, or critical CHD in her family. No mother had a USG report suspecting critical CHD in the foetus or was advised to undergo foetal echocardiography. A history of consanguineous marriages was reported in 6.9% of the parents. Table 1 shows the distribution of maternal characteristics.

**Table 1:** Distribution of Maternal Characteristics

Maternal characteristics	Group	Frequency	Percentage
**Maternal Age (years)**	≤ 20	108	10.0
	21 to 25	597	55.2
	26 to 30	342	31.6
	> 30	35	3.2
**Gestational Age (weeks)**	37	97	9
	38	344	31.7
	39	348	29.4
	40	217	20.1
	41	106	9.8
**Registration Status**	Non-registered	425	39.3
	Registered	657	60.7
**H/O Diabetes**	No	1,079	99.7
	Yes	3	0.3
**Mode of Delivery**	Normal	842	77.8
	LSCS	240	22.2
**Infections during pregnancy**	No	1,045	96.6
	URTI	19	1.8
	Malaria	8	0.7
	Torch	6	0.6
	Hepatitis	4	0.4
**Total**		1,082	100.0

All neonates cried immediately after birth. Table 2 shows the neonatal characteristics, and Table 3 shows group-wise distribution of the neonates in the study.

**Table 2:** Characteristics of the neonatal study population.

Neonatal characteristics	N	Mean ± SD	Range
**Maternal Age (years)**	1,082	23.54 ± 2.80	19–32
**Gestational Age (weeks)**	1,082	39.41 ± 1.33	37–41
**Hours of Life**	1,082	8.30 ± 2.85	4–22
**Birth Weight (Kg)**	1,082	2.72 ± 0.124	2.5–3.2
**Heart Rate (beats per minute)**	1082	151 ± 13	120–168
**Respiratory Rate (rate/ min)**	1,082	43.5 ± 5	38–58
**SpO_2_ (in %) First Reading (R_1_)**	1,062	97 ± 1	95–100
	18	93 ± 1	92–94
	2	86 ± 1	85–87
**SpO_2_ (in %) Second Reading (R_2_)**	15	96 ± 1	96–100
	3	93 ± 1	92–94
**SpO_2_ (in %) Third Reading (R_3_)**	15	97 ± 1	96–100
	3	93 ± 1	92–94

**Table 3:** Group-wise distribution of neonates in the study.

	Groups	Frequency	Percentage
**Birth Weight**	2.5 to 3 Kgs	1,021	94.4
	3.1 to 4 Kgs	61	5.6
**Sex**	Female	513	47.4
	Male	569	52.6
**Heart Rate (beats / min)**	120 to 140	183	16.9
	141 to 160	733	67.7
	161 to 180	166	15.3
**Respiratory Rate (per minute)**	38 to 47	821	75.9
	48 to 58	261	24.1
**Hours of Life at Pulse oximetry Evaluation**	2 to 6 hours	324	29.9
	6 to 12 hours	693	64.0
	12 to 24 hours	65	6.0
**Total**		1,082	100.0

After detailed physical examination, critical CHD was suspected in 7 out of 1,082 neonates (0.6%). After pulse oximeter screening, five infants (0.5%) were detected with low SpO2 readings, and thus echo was done (Table 4). The mean number of hours of life at the time of POx measurement in 324 neonates evaluated for between 2–6 hours was 5 ± 0.47 hours while neonates evaluated between 12–24 hours of life had a mean life of 17 hours.

**Table 4:** Oxygen Saturation (SpO_2_) by Pulse Oximeter

SpO_2_	Groups	Frequency	Percentage
1^st^ Reading (R_1_)	≥ 95%	1,062	98.1
	90%–94%	18	1.7
	< 90%	2	0.2
	**Total**	**1,082**	**100.0**
1^st^ Repeat (R_2_)	≥ 95%	15	83.3
	90%–94%	3	16.7
	**Total**	**18**	**100.0**
2^nd^ Repeat (R_3_)	≥ 95%	15	83.3
	90%–94%	3	16.7
	**Total**	**18**	**100.0**

Out of 1,082 neonates screened, critical CHD was diagnosed in 5 asymptomatic neonates, and thus a prevalence of 0.5% was found. Of those 5 cases diagnosed by echo, 3 had cyanotic and 2 had acyanotic CHD. Cyanotic CHD diagnosed was Tetralogy of Fallot (TOF), TGA with ventricular septal defect (VSD), and Double Outlet Right Ventricle (DORV) without Pulmonary stenosis (PS). Acyanotic CHD diagnosed was Atrial Septal Defect (ASD) with Pulmonary Hypertension (PH) and PDA. There were 3 (60%) females and 2 (40%) males among the critical CHD cases analysed. A total of 4 out of 5 newborns (80%) having critical CHD were detected by POx with abnormal oxygen saturation. Table 5 represents the oxygen saturation as well as the neonatal presentation of the critical CHD cases suspected.

**Table 5:** Saturation and presentation of newborns suspected of having critical CHD.

Sr. No	SpO_2_ (%)	Subsequent SpO_2_ (%)	Neonatal Presentation	Suspected By	2D ECHO Diagnosis
1	87	90	Grade 3, Systolic murmur	POx and Clinical Examination	TGA + VSD
2	85	87	Grade 4, Ejection systolic murmur at left upper parasternal region	POx and Clinical Examination	TOF
3	92	92	Normal	Pox	DORV
4	92	92	Normal	Pox	ASD + PH
5	95	96	Bounding pulse, Grade 4, continuous murmur, left infra-clavicular region	Clinical Examination	PDA
6	94	94	Asymptomatic	POx	Normal
7	100	–	Grade 2 systolic murmur	Clinical Examination	Normal
8	98	–	Grade 2 systolic murmur	Clinical Examination	Normal
9	97	–	Grade 2 systolic murmur	Clinical Examination	Normal
10	100	–	Grade 2 systolic murmur	Clinical Examination	Normal

Out of 5 newborns diagnosed with CHD, 3 (60%) were born to mothers from 20 to 25 years of age while mothers aged 25–30 years and over 30 years of age had 1 case in each group. Mean maternal age in positive cases was 26.2 ± 3.7 years. Overall, 40% [2] of the 5 CHD cases were delivered at a GA of 37 weeks, 40% had 38 weeks gestation, and in 20% of the cases GA was 39 weeks. In all, 3 newborns with critical CHD were registered for antenatal care while 2 did not have maternal registrations.

Two newborns (40%) were evaluated between 6 to 12 hours of life, and 3 (60%) were evaluated after 12 hours. Four newborns (80%) weighed between 2.5 to 3 kg while only 20% weighed more than 3 Kg. TOF was detected at an age of 8 hours by POx, and the neonate had a birth weight of 2.7 kg. A neonate with TGA + VSD was screened by POx at 12 hours of age and had a birth weight of 2.6 kg. ASD with PH was suspected at an age of 14 hours. Neonates with DORV and PDA were screened by POx at 18 hours of life. The birth weights of neonates with DORV, PDA, and ASD with PH were 3.2 kg, 2.6 kg, and 2.7 kg, respectively. The mean birth weight of neonates at the time of screening was 2.7 ± 0.25 kg and the median age of diagnosis was 12 hours.

## Discussion

The current study examined the utility of POx as a screening test for the detection of critical CHD in asymptomatic newborns. Out of 1,082 neonates screened, 5 cases of critical CHD were confirmed by echocardiography. The incidence of critical CHD was 0.5% (5/1,000 live births). The overall prevalence of CHD in this study is similar to that observed by Kapoor et al. [29], lower than that observed by Vaidyanathan et al. in a hospital-based study [30], and equivalent to the current status report of CHD in India [5].

In the current study, pulse oximeter readings of less than 95% were found in 0.5% neonates that required echo while 99.5% neonates had normal SpO_2_ with a mean of 97%. An earlier study by Hoke et al. reported that 0.02% study population showed abnormal POx tests and no significant difference was observed concerning birth weight, GA, or gender between the normal and abnormal groups [24]. A study based in Mexico in 2015 also correlated well with our study [31]. With respect to diagnosis of critical CHD in neonates detected with low POx values, Arletazz et al. reported that low POx values were detected in 0.7% neonates only, out of which 71% were diagnosed to have critical CHD [32]. Barreto et al. in 2019 have reported that 76.3%–82% of cases screened by POx, had critical CHD [33]. In our study, out of five neonates having low POx readings, four (80%) were confirmed to have critical CHD. It has been reported that with the introduction of POx in West Gotaland 92% of the babies with duct-dependent circulation were diagnosed before leaving the hospital [20]. Pure left-to-right shunts such as VSD, ASD, or PDA cannot be detected by pulse oximetry, but some of these defects have proved to be screen positive. This was observed to happen almost all the time within the first 24 hours of life according to Arlettaz [32]. The probable cause for it was thought to be bidirectional shunting during early postnatal pulmonary hypertension [34].

Physical examination alone in this study generated seven referrals for echo (Figure 2), although in four of these cases echo findings were normal. These four cases had innocent murmurs with normal pulse oximetry screening; however, we performed echo to rule out CHD on the discretion of treating physician. In a study by Vaidyanathan et al., 2.9% of cases had a positive clinical evaluation with the most common criteria being murmur in 1.6% of the cases [30]. According to de-Wahl and colleagues, the detection rate of CHD by physical examination alone was 62.5%, which is quite similar to our study, with a detection rate of 60% [20]. A physical examination has been proved to be an inadequate screen for CHD by various other studies as well [35, 36]. An asymptomatic newborn may appear pink despite having clinically significant desaturation, although on examination a murmur can predict CHD. A study by Arlettaz *et al*. has reported that 73% of infants with CHD had a murmur at the time of echo. Only 35% of cyanotic CHD babies present with a murmur, whereas all non-cyanotic CHD have been detected through a murmur [32]. These results confirm the importance of clinical examination but at the same time also show that the presence of murmur does not correlate well with the severity of cardiac lesions. Moreover, detection of a murmur depends on the examiner’s skill and experience, the timing, the frequency of examination, and the conditions under which the examination takes place. These may be the reasons for the variation of reported prevalence of murmur in neonates as shown by different studies [35, 36].

Detection of critical CHD by POx screening is better than physical examination alone. Combined clinical examination and POx could detect all the critical CHD cases [37, 38, 39]. Of five CHD cases detected and confirmed by echo, four were detected by pulse oximetry and three by clinical examination. An ECG is specific and precise for the definition of heart rhythm disturbance and is just suggestive for structural abnormalities and myocardial dysfunction [16, 40, 41]. In our study, cyanotic CHD diagnosed was TOF, TGA with VSD, and DORV without PS. Acyanotic CHD diagnosed was ASD with PH and PDA. In a previous study, it was found that POx screening is useful in detecting cyanotic CHD, critical duct-dependent systemic lesions, and PPHN in sick neonates [42]. Certain cardiac lesions present with some degree of neonatal hypoxemia and include proximal aortic arch anomalies, COA with PDA, Ebstein’s anomaly, DORV, and single ventricle lesions. These are considered to be secondary screening targets for POx [43].

The sensitivity of POx for detecting CHD, as reported by Wahl GA et al. was 62% and by physical examination alone was 63%. Combining both had a higher sensitivity of 82.76% as compared with either of the other methods [20]. In a hospital-based study by Vaidyanathan B et al., POx and clinical evaluation had low sensitivity (less than 20%) in the detection of critical CHD [30]. A sensitivity of 76.3% and specificity of 99.9% were reported by both Barreto et al. and Plana et al. [33,44]. Arlettaz et al. found a specificity of 99.7%, positive predictive value (PPV) of 63%, and negative predictive value (NPV) of 100% in diagnosing cyanotic CHD by POx [32]. Therefore, previous literature reported that high sensitivity of POx detects critical CHD and thus supports its utilisation as a screening test in otherwise asymptomatic neonates. Notably, the false positive rate observed by Wahl GA et al. (0.17%) [20] and Plana et al. [44] was much lower. Wahl GA et al. analysed various studies on POx screening, and when data were restricted to critical left-sided obstructive lesions, they observed sensitivity values of 0%– 50% and false-positive rates between 0.01%–12% in the asymptomatic population [20]. In contrast, the report of the Tennessee Task Force on screening newborn infants for CHD had questioned the utility of oximetry screening because of unclear false-positive rates of the screening program [7, 21].

Our study had certain limitations. It was conducted in resource-limited settings, and thus, we could not use the reference standard, echocardiography, for all the participants. It was performed only on those neonates who had shown abnormal pulse oximetry or clinical examination findings. Hence, we could not calculate the sensitivity, specificity, NPV, or PPV. Moreover, the sample size was limited, and this made the calculation of the effectiveness of POx screening difficult.

## Conclusion

In the absence of clinical findings, newborns with critical cardiac malformations can also be discharged. Critical CHD missed by the clinical examination alone can be detected by pulse oximetry in neonates. Pulse oximetry is a simple, non-invasive, feasible, reliable, and cost-effective screening test to detect critical CHDs during early neonatal life. With early detection of CHD, appropriate medical intervention can be begun promptly, so that there is less risk of developing severe symptoms or complications, and this therefore may lead to a better outcome.

## Figure Titles and Captions

**Figure 1.** Flow chart showing POx value and progression of investigations.

**Figure 2.** Association of pulse oximetry, neonatal evaluation and echocardiography in critical CHD.

POx: Pulse Oximetry, ‘+’ **critical** CHD Suspected, ‘–’ **critical** CHD not suspected, TGA: Transposition of Great Arteries, VSD: Venticular Septal Defect, DORV: Double Outlet Right Ventricle (subaortic VSD without pulmonary stenosis), TOF: Tetrology of Fallot, ASD: Atrial Septal Defect, PDA: Patent Ductus Arteriosus, PH: Pulmonary Hypertension.
